# Enhancement of Cerenkov Luminescence Imaging by Dual Excitation of Er^3+^, Yb^3+^-Doped Rare-Earth Microparticles

**DOI:** 10.1371/journal.pone.0077926

**Published:** 2013-10-25

**Authors:** Xiaowei Ma, Fei Kang, Feng Xu, Ailing Feng, Ying Zhao, Tianjian Lu, Weidong Yang, Zhe Wang, Min Lin, Jing Wang

**Affiliations:** 1 Department of Nuclear Medicine, Xijing Hospital, Fourth Military Medical University, Xi’an, PR China; 2 The Key Laboratory of Biomedical Information Engineering of Ministry of Education, School of Life Science and Technology, Xi'an Jiaotong University, Xi'an, PR China; 3 Bioinspired Engineering and Biomechanics Center, Xi'an Jiaotong University, Xi'an, PR China

## Abstract

Cerenkov luminescence imaging (CLI) has been successfully utilized in various fields of preclinical studies; however, CLI is challenging due to its weak luminescent intensity and insufficient penetration capability. Here, we report the design and synthesis of a type of rare-earth microparticles (REMPs), which can be dually excited by Cerenkov luminescence (CL) resulting from the decay of radionuclides to enhance CLI in terms of intensity and penetration. **Methods**: Yb^3+^- and Er^3+^- codoped hexagonal NaYF_4_ hollow microtubes were synthesized via a hydrothermal route. The phase, morphology, and emission spectrum were confirmed for these REMPs by power X-ray diffraction (XRD), scanning electron microscopy (SEM), and spectrophotometry, respectively. A commercial CCD camera equipped with a series of optical filters was employed to quantify the intensity and spectrum of CLI from radionuclides. The enhancement of penetration was investigated by imaging studies of nylon phantoms and nude mouse pseudotumor models. **Results**: the REMPs could be dually excited by CL at the wavelengths of 520 and 980 nm, and the emission peaks overlaid at 660 nm. This strategy approximately doubled the overall detectable intensity of CLI and extended its maximum penetration in nylon phantoms from 5 to 15 mm. The penetration study in living animals yielded similar results. **Conclusions**: this study demonstrated that CL can dually excite REMPs and that the overlaid emissions in the range of 660 nm could significantly enhance the penetration and intensity of CL. The proposed enhanced CLI strategy may have promising applications in the future.

## Introduction

Nuclear imaging allows for sensitive and noninvasive measurement of radionuclide-labeled probes in living animals and humans [1]. However, wider application of nuclear imaging is limited by the necessity for long acquisition time and expensive instruments [2]. Although optical imaging is of much lower expense and higher throughput, it is still limited by the paucity of available imaging agents for clinical use, with only three non-specific agents approved by the US Food and Drug Administration (FDA): indocyanine green (ICG), methylene blue, and fluorescein [3]. Cerenkov luminescence (CL) is an intrinsic optical signal generated when a charged particle travels through a medium faster than the velocity of light in that medium. Because many radionuclides (e.g. ^131^I, ^18^F) approved by the FDA for clinical use are emit charged particles, capable of producing CL that can be detected by low-cost charge-coupled device (CCD) cameras [4,5]. The concept of Cerenkov luminescence imaging (CLI) provides a potential method to achieve multimodality molecular imaging by combining radionuclide labeled probes and optical imaging together [6]. Since 2009, CLI has been successfully utilized in various fields of preclinical study, including *in vivo* tumor imaging [5,7,8], therapy monitoring [9], intra-operative guidance [10], lymphography [11], endoscopy [3,12] and *in vivo* 3-dimensional reconstruction [13-15].

Despite these notable advancements in CLI, the use of CLI is highly restricted by its relatively weak luminescent intensity and insufficient tissue penetration capability [2,7,16]. This could mainly be attributed to the spectral characteristics of CL. For example, the CL spectrum is continuous, and the most intensive CL is in the spectrum of short wavelengths (ultraviolet/blue) below 650 nm, which can be easily scattered and absorbed by biological tissues [2,17]. The region of the CL spectra from 650 nm to 900nm is very weak although it has better penetration ability in biological tissues. Additionally, the regions of the CL spectra below 500 nm and over 900 nm are out of the maximally effective detection range of common CCD cameras, according to the user’s manuals for commercial CCD camera. Because of those reasons, the natural distribution of the CL spectra is not perfectly suitable for *in vivo* imaging, especially for the detection of deeply seated targets. Thus, to summarize, the preferred emission band for Cerenkov luminescence, taking both penetration and intensity into consideration, is within the narrow range from 650 to 900 nm.

Previous studies have indicated that the coupling of CL with other fluorophore (e.g., small molecules, quantum dot (QD)) was able to transform some of the blue-weighted CL spectra to red-shifted emissions [18-20]. In this scenario, CL serves as the energy donor, while the fluorophores represents the energy acceptor. Such a strategy may be of great significance for the development of CLI technologies with enhanced intensity and penetration. Considering the intensity of CL is relative to the inverse square wavelength while the longer wavelength light has better penetration and lower absorption by tissue, a material with large Stock-shift should be better to convert much shorter wavelength CL to longer wavelength luminescence. Rare-earth nanoparticles (RENPs) with advantaged properties such as high photostability, absence of blinking, large Stoke-shifts, long lifetimes and low cytotoxicity are promising for such CL conversion strategy [21].

 RENPs are reported to simultaneously possess both down-conversion and up-conversion effects [22]. Down-conversion is a process through which higher energy photons are absorbed, while lower energy photons are emitted [23]. In contrast, the up-conversion effect involves of emission of higher energy photons through sequential absorption of lower energy photons [24]. Therefore, RENPs possessing these two effects are capable of being dually excited by either the ultraviolet/blue spectrum or the near-infrared (NIR) spectrum. Moreover, emissions resulting from the two excitation sources could be adjusted to overlay in a desirable wavelength range for *in vivo* imaging by modifying the proportion of excitation sources within the RENPs [25]. Such dual excitation characteristics of RENPs indicate possible enhanced emission resulting form excitation by both the blue-weight band and the NIR band of CL.

In this study, we aimed to design and synthesize a novel type of REMPs, NaYF_4_:Er^3+^, Yb^3+^ hollow microtubes, which could be simultaneously dually excited by the CL spectrum below 650 nm (520 nm) and over 700 nm (980 nm). The emission bands from the dual excitation were adjusted to be overlaid in the desirable range of 650-900 nm for *in vivo* imaging. The feasibility of this dual-excitation-based enhancement strategy was then evaluated on phantoms and pseudotumor models. Our findings offer an alternative route for exploring the possible further applications of CLI with enhanced emission intensity and tissue penetration.

## Materials and Methods

### Materials

The radionuclides ^18^F and ^131^I were obtained in the form of 2-18-fluoro-2-deoxy-D-glucose (^18^F-FDG) and Na^131^I. ^18^F-FDG was produced by cyclotron (GE Industries Inc., USA) and FDG reagent kit (ABX, Germany). Na^131^I was purchased from Chengdu Gaotong Isotope Co., Ltd. (China). For the synthesis of REMPs, NaF was purchased from Tianjin Yong Sheng Fine Chemical Co., Ltd. Y(NO_3_)_3_, Yb(NO_3_)_3_ and Er(NO_3_)_3_ were purchased from Alfa Aesar (UK). All chemicals were analytical-grade reagents and were used without further purification.

### Synthesis and characterization of REMPs

NaYF_4_ microparticles codoped with Yb^3+^ and Er^3+^ ions were synthesized by a hydrothermal method. In a typical procedure, Y(NO_3_)_3_, Yb(NO_3_)_3_, and Er(NO_3_)_3_ solutions with a molar ratio of 80:18:2 were added into beaker and mixed by stirring. Then, 50 mmol sodium fluoride dissolved in 10 mL ultrapure water was added to the above solution with a Ln^3+^(Y^3+^, Yb^3+^, Er^3+^):NaF molar ratio of 1:16. Subsequently, the solution was stirred for 10 min. The pH value of the solution was then adjusted to around 3.0 by using dilute HNO_3_ and NH_3_·H_2_O solutions. The mixture was then transferred into a 50-mL Teflon vessel. The vessel was tightly sealed in an autoclave, heated at 180°C for 14 h, and then naturally cooled down to room temperature. The products were washed and centrifuged for 3 times using ethanol and deionized water. After drying in a vacuum oven for 12 h, the final white powders were collected for further use. Scanning electron microscopy (SEM; FEI Quanta 200, Philips, Netherlands) was utilized to identify the size and morphology of REMPs. The crystal phase of REMPs was characterized by an X-ray diffractometer (XRD, Bruker D8 Discover, USA). The absorption spectrum of REMPs with a concentration of 0.025 mg/mL was recorded with a UV-VIS-NIR absorption spectrophotometer (Cary 500, Varian, USA). To determine the emission spectrum of REMPs excited by external sources with wavelength of 520 and 980 nm, the emission spectrum of the REMPs were measured respectively by using a fluorescence spectrophotometer (Edinburgh Instruments, Britain). 520 nm excitation source was generated by the optical grating of the spectrophotometer, and the 980 nm one was from a laser diode. All spectral measurements were performed at room temperature.

### Measurement of emission spectrum excited by CL

Radioactive sources containing 3.7 MBq of Na^131^I or ^18^F-FDG with or without 2 mg/mL REMPs dissolved in dimethylsulfoxide (DMSO) with a final volume of 200 μL were prepared in 96-well black plates (Nunc, USA). The emission spectrum of REMPs excited by CL was measured by IVIS System (Caliper Life Science, USA) equipped with 18 filters ranging from 500 nm to 840 nm, with a 20-nm interval in full width at half maximum. The concentration of the added REMPs was based on the tested favorable ratio of radionuclide and microparticles [26]. Optical images were collected for 20 sec and were further analyzed using the region of interest (ROI) method. The same procedure was repeated for 3 times.

### Assay of the enhancement of CLI intensity

CL excites a secondary source of emission from REMPs, which may improve the intensity of CLI. To investigate the enhanced CLI intensity, samples containing Na^131^I, REMPs, or both Na^131^I and REMPs were added to 3 wells of a 96-well black plate with a final volume of 200 μL as follows: 2 mg/mL REMPs, 3.7 MBq Na^131^I, or 2 mg/mL REMPs with 3.7 MBq Na^131^I. ^18^F-FDG as the parallel group, and ^99m^Tc as the negative control group, were similarly prepared as above. Optical images were detected using IVIS system for 60 sec. The same procedure was repeated for 6 times. Total CLI intensity was measured by drawing ROIs along the wall of each well. 

### Assay of the enhancement of CLI penetration

 To demonstrate the enhancement of CL penetration capacity, we performed a cubic nylon phantom experiment. The optical properties of the homogeneous nylon phantom were the same as that of mouse lungs. Two holes with a diameter of 2 mm and the small distance from its center axel to the top surface of the phantom were drilled into the phantom to embed rubber capillary tubes with the same scattering effect of the phantom. A series of depths from the top surface of the phantom to the center axel of the hole (0, 2.5, 5.0, 7.5, 10, and 15 mm) was tested. 3.7 MBq ^18^F-FDG in a volume of 20 μL was injected at the bottom of a rubber capillary tube, and 3.7MBq ^18^F-FDG with 2 mg/mL REMPs with a final volume of 20 μL was injected into another tube. The 2 tubes were painted black except the side facing the camera to avoid the interaction of the CL from each other. Then the two tubes were placed into the holes of the phantoms. Optical images were detected using IVIS system for 60 sec. The same procedure was repeated for 6 times. 

### Pseudotumor Study

To demonstrate the comprehensive enhancement capacity of REMPs on the real biological organism, we followed a pseudotumor study on living animals as described in previous studies [18,19,27]. Animal care and protocols were approved by the Fourth Military Medical University Animal Studies Committee (Protocol 20090260). All animal procedures were performed under anesthesia by inhalation of a 1%–2% isoflurane-oxygen mixture. 50 μL Matrigel (BD Biosciences, USA) was mixed with either 50 μL REMPs dissolved in DMSO or 50 μL pure DMSO in a microfuge tube. Then, 3.7 MBq ^18^F-FDG was separately added in the microfuge tubes to achieve a total volume of 150 μL and a final REMPs concentration of 2 mg/mL. Anesthetized nude mice were injected subcutaneously with 100 μL of the Matrigel+REMPs+^18^F-FDG mixture in the right flank and 100 μL of the Matrigel+DMSO+^18^F-FDG mixture in the left flank. Mice were kept in warm for 5 min until the Matrigel solidified. The mice were then imaged in a microPET/CT (Mediso Ltd., Hungary) to assure the radioactive sources of each pseudotumor were roughly the same. Optical imaging was performed on IVIS system for 60 sec after finishing PET/CT scans. The total optical signal value of each pseudotumor was calculated by ROI method. The radioactivity in each pseudotumor was calculated based on PET/CT imaging by using the 3D-ROI method. The optical signal value was normalized by the radioactivity in the same pseudotumor. 

### Statistical analysis

Data were reported as the mean ± SEM. Pairs were compared by Student’s t-tests, and *p*-values of less than 0.05 were considered significant.

## Results

### Characterization of REMPs

**Figure 1:**
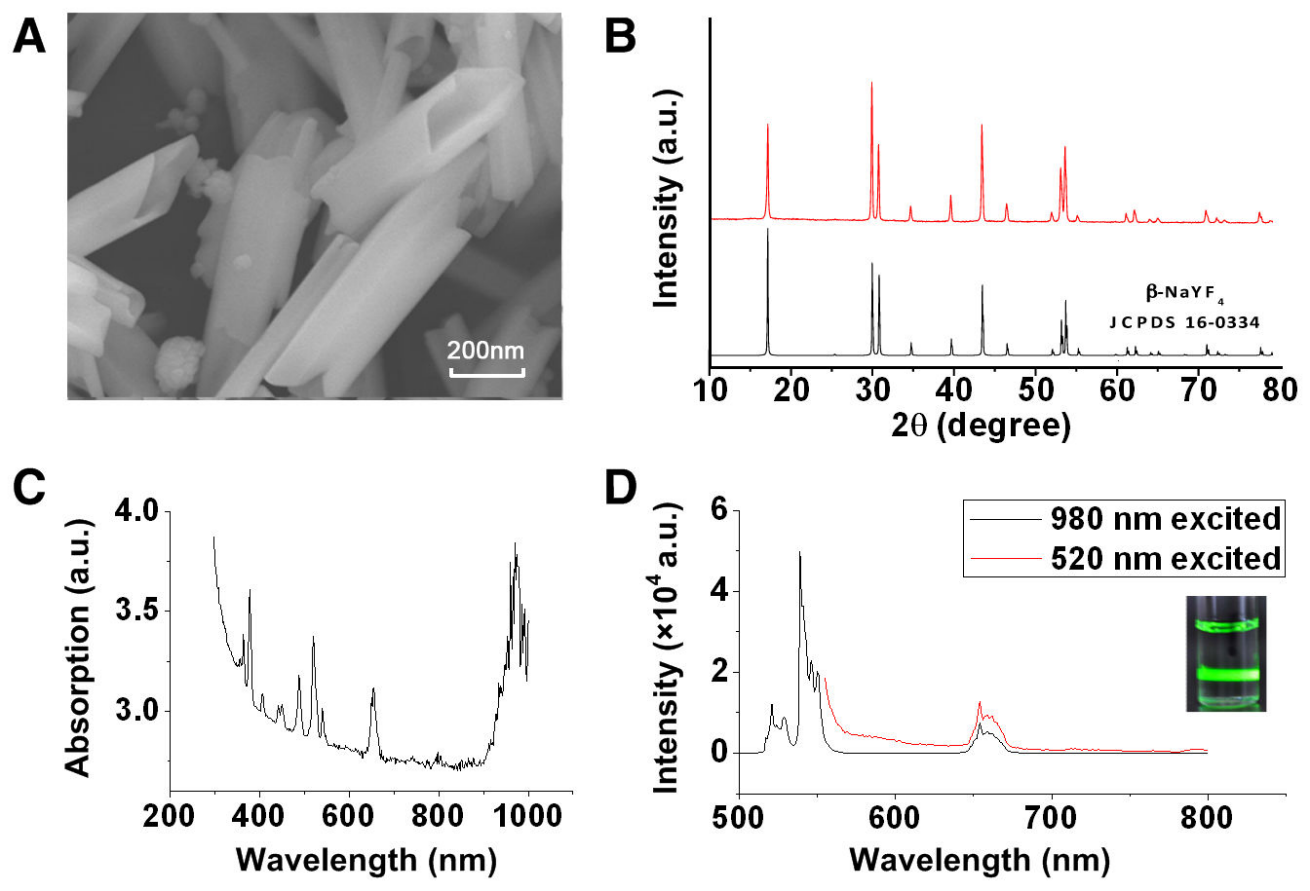
Characterization of REMPs. (A) SEM image of NaYF_4_:Yb^3+^,Er^3+^ nanoparticles. (B) XRD pattern of NaYF_4_:Yb^3+^,Er^3+^ microparticles and standard pattern. (C) UV-IR absorption spectrum of NaYF_4_:Yb^3+^,Er^3+^ microparticles with a concentration of 0.025 mg/mL. (D) Fluorescence emission spectra of NaYF_4_:Yb^3+^,Er^3+^ microparticles under excitation with 520 nm and 980 nm lasers. Inset: photo of NaYF_4_:Yb^3+^,Er^3+^ microparticles dissolved in DMSO under 980 nm laser excitation.

A representative SEM image of NaYF_4_:Yb^3+^,Er^3+^ microparticles is shown in Figure 1A, which illustrated that the synthesized microcrystals were hollow tubes. We observed that the microtube with an average size of 133±28 nm × 467±72 nm (diameter × length). XRD patterns of the as prepared microparticles are shown in Figure 1B. As can be seen, these microparticles mainly had a hexagonal structure of NaYF_4_, which agreed well with the standard pattern (JCPDS 16-0334). The UV-NIR spectrum of the NaYF_4_:Yb^3+^,Er^3+^ microparticles (Figure 1C) showed strong absorption and characteristic absorption peaks of 380, 520, 640, and 980 nm. This makes it a potential energy acceptor to absorb the 520 and 980 nm luminescence of CL. The emission spectrum of NaYF_4_:Yb^3+^,Er^3+^ microparticles is shown in Figure 1D. Under excitation of 980 nm, the up-conversion emission spectral distribution was between 500 and 800 nm, with peaks at 520, 540, and 660 nm. Under excitation of 520 nm, the down-conversion emission spectrum of NaYF_4_:Yb^3+^,Er^3+^ microparticles was between 550 and 800 nm, with a peak at 660 nm. The inset in Figure 1D shows that the synthesized microparticles emitted strong green fluorescent light following excitation by the 980 nm laser with power density of 250 mW.

### Measurement of emission spectrum excited by CL

**Figure 2:**
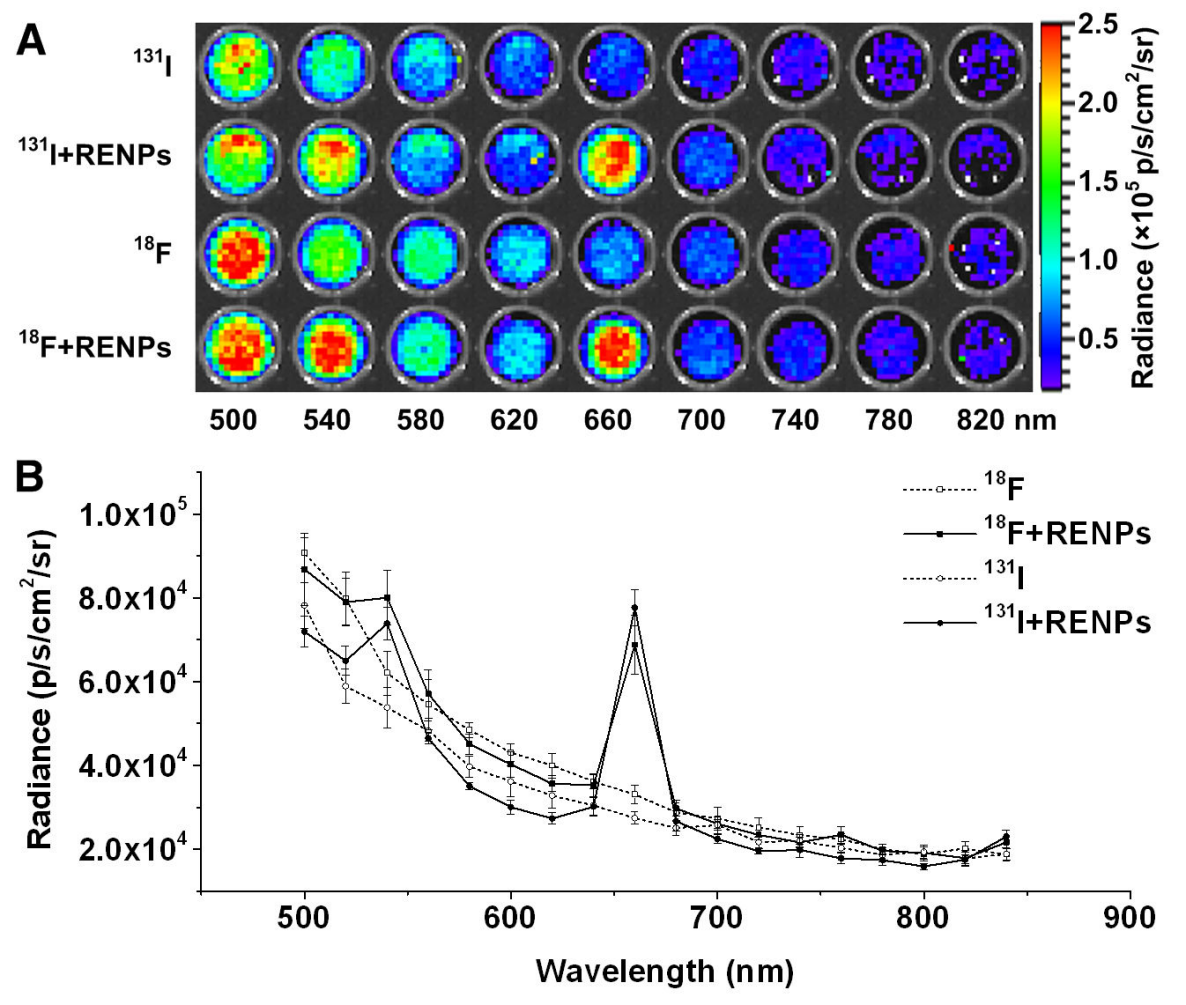
Spectra analysis. (A) CLI of 3.7 MBq Na^131^I and ^18^F-FDG with or without REMPs using a narrow band emission filter (from 500 to 840 nm, 20 nm bandwidth). (B) Cerenkov luminescence spectra of 3.7 MBq Na ^131^I and ^18^F-FDG with or without REMPs drawn according to the intensity using different filters. When mixed with REMPs, both Na^131^I and ^18^F-FDG shared similar spectral distributions and two significant intensity peaks at 540 and 660 nm.

For the CLI tested, the imaging of the luminescence at different wavelengths using narrow band filters is shown in Figure 2A, and the spectral distribution is shown in Figure 2B. Both Na^131^I and ^18^F-FDG shared a similar distribution of CL. When mixed with REMPs, both of these two nuclides shared another similar spectral distribution, and significantly increased intensity peaks at 540 and 660 nm were identified.

### Assay of the enhancement of CLI intensity

**Figure 3:**
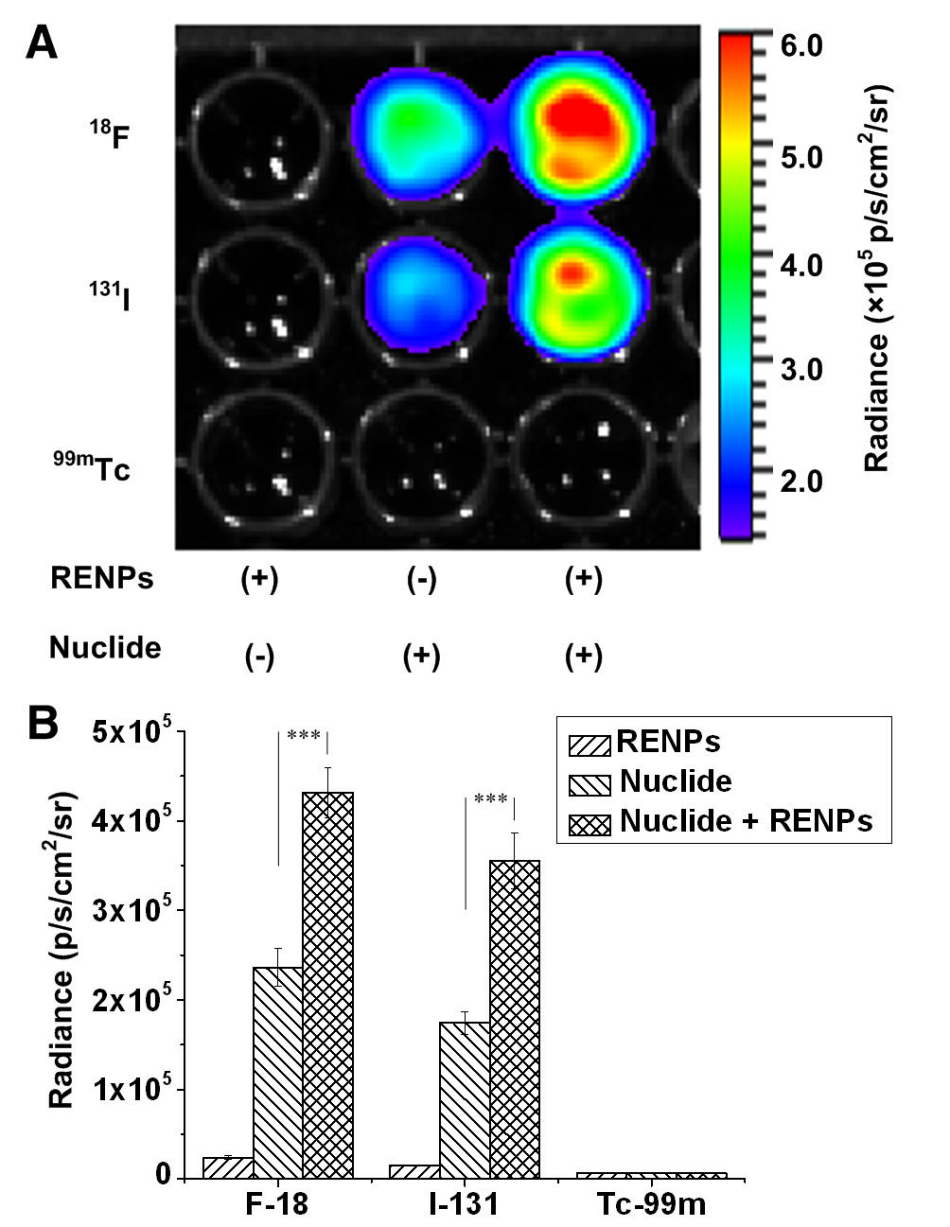
CLI intensity assay. (A) CLI of 96-well black plates containing 2 mg/mL REMPs, 3.7 MBq radionuclides (^18^F-FDG, Na ^131^I, and ^99m^Tc), or both per well in a final volume of 200 μL. ^99m^Tc was used as the negative control for CLI. (B) Quantification analysis of radiance according to different radionuclides with or without REMPs. The luminescence intensity of the radionuclide mixed with REMPs was significantly enhanced (***P < 0.005).

As shown in Figure 3A and 3B, the detected luminescence intensity was almost twice as enhanced in the ^18^F-FDG or Na^131^I samples containing REMPs, as compared with samples of pure ^18^F-FDG or Na^131^I (*p* < 0.001). No emission, but background noise, was observed in samples of pure REMPs, indicating that the REMPs themselves could not be excited by γ rays. Since ^99m^Tc emitted γ photons with energies lower than the threshold for producing CL [7], the wells containing ^99m^Tc failed to generate CL and thus failed to excite REMPs.

### Assay of the enhancement of CLI penetration

**Figure 4:**
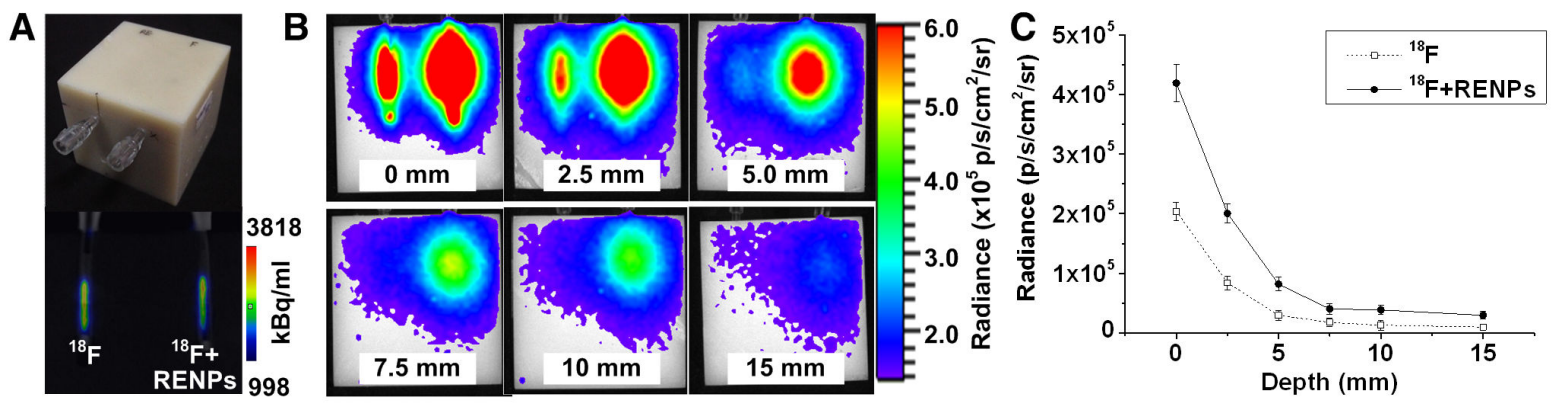
CLI penetration assay performed with nylon phantoms. (A) The top part is the photo of the phantom. The bottom part is the PET/CT imaging of the phantom. The radioactivity of the 2 samples were the same. (B) CLI for the penetration of 3.7 MBq ^18^F-FDG with (right) or without (left) REMPs, as imaged using a CCD camera. The numbers indicate the distance from the source center to the surface of the phantom. (C) Quantitative analysis of the phantom studies. The broken line with hollow dots and the line with solid dots show the relationship between the penetration depth and the intensity of ^18^F-FDG alone or ^18^F-FDG with REMPs, respectively.

MicroPET/CT scans revealed that the radioactivity of the two radiation sources were roughly the same (Figure 4A). The results of optical imaging showed that the emission intensity of both ^18^F-FDG with REMPs and ^18^F-FDG alone decreased with increasing depth from the top surface of the phantoms to the center axel of the holes. The maximum penetration of ^18^F-FDG with REMPs reached up to 15 mm, while ^18^F-FDG alone had a maximum penetration of around 5 mm (Figure 4B and 4C). 

### Pseudotumors Study

**Figure 5:**
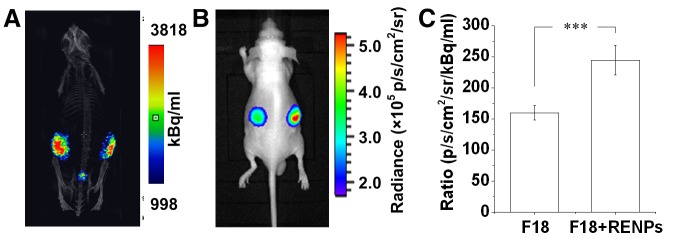
*In* *vivo* CLI of the subcutaneous Matrigel pseudotumor animal models. (A) MicroPET/CT imaging of subcutaneous Matrigel pseudotumors containing 37 MBq ^18^F-FDG (left flank) or ^18^F-FDG with 2 mg/mL REMPs (right flank) in mice. (B) CLI of the same subcutaneous Matrigel pseudotumor mouse model. (C) Quantitative analysis of the Cerenkov luminescence intensity of the pseudotumors on both sides. The pseudotumor of ^18^F-FDG with REMPs showed stronger luminescence intensity than that of ^18^F-FDG with DMSO (***P < 0.001).

The radioactivity of the pseudotumors detected by microPET/CT was demonstrated to be similar in both pseudotumors with or without REMPs (Figure 5A). Optical images showed an enhanced intensity on the right flanks of the mice (Figure 5B). Using the nuclear signal as a reference, we observed that the relative intensity of the REMPs mixed with ^18^F-FDG injected pseudotumors was significantly higher than that of the ^18^F-FDG injected pseudotumors (244.7 ± 23.5 vs. 159.9 ± 11.6; n = 6, *p* < 0.001; Figure 5C).

## Discussion

In this study, we found that the Er^3+^,Yb^3+^-doped REMPs has two main absorption peaks on 520 nm and 980 nm and main emission peak on 660 nm which was within the desirable range for CCD detection and for penetrating biological tissues. And when mixed with ^18^F-FDG or Na^131^I, the photon emission peak at 660 nm was also observed as we expected. It could be considered as the REMPs were dually excited by the partial light of CL around 520nm and 980nm because the spectrum of CL covers the absorption spectra of REMPs. But as we known, those two radionuclides we tested in this work both emit high-energy charged particles, and we are not sure whether the high energy charged particles have any effect on REMPs or not. We hypothesis that the excitation mechanism for our experimental case may also involves non-radiation resonance energy transfer process. During this process, the nuclides (^131^I and ^18^F) act as energy donor while the REMPs act as energy acceptor. So, when nuclides and REMPs get closed to each other (e.g., mixture), non-radiation energy transfer may occur and generate specific emission. This will be further studied in our future works. Whatever, this strategy approximately doubled the overall detectable intensity of CLI and extended its maximum penetration in nylon phantoms about 3-fold. The penetration study in living animals indicated a similar result.

RENPs doped with rare-earth activator ions and rare-earth sensitizer ions possess a unique optical property known as photon up-conversion. Such a unique property gives them several advantages in bioimaging, including remarkable penetration depth into tissues upon NIR excitation [28], significantly decreased autofluorescence [29], no photobleaching or photoblinking, and high spatial resolution during bioimaging [30,31]. In addition to photon up-conversion, these nanoparticles or microparticles simultaneously possess the capability of photon down-conversion [32], giving them dual excitation characteristics under an excitation source having both UV and NIR light. In fact, the concept of combining up-conversion and down-conversion to achieve dual excitation effects has already been proposed and tested [25,33]. Apart from their superior optical properties, RENPs also exhibit low cytotoxic to a broad range of cell lines [28,34-36].

In previous studies, enhanced CLI could be achieved by utilizing QDs as energy acceptors [18-20,27]. The principles of this enhancement were based on the down-conversion effect of QDs by transferring the excitation of a short wavelength from CL into the emission of a longer wavelength. Liu et al., Gelovani et al., and Capenter et al. clarified such QDs-based enhancement strategies and intuitively improved the penetration of CLI by using pseudotumors mouse models [18,19,27]. In their studies, the penetration of CL could be enhanced by transferring the excitation in the blue-weighted spectrum to an emission in the range of 629–705 nm. Using a similar mechanism, we demonstrated, in this proof-of-concept study, that Er^3+^,Yb^3+^-doped REMPs, with both down-conversion and up-conversion effects, could not only be excited by the blue-weight spectrum of CL as the QD, but could also transfer the undetectable, longer-wavelength more than 900 nm CL spectrum into a peak at 660 nm, thus extending the excitation source and adding additional intensity to CLI.

CLI has recently been recognized as a potential optical imaging modality [16]. However, the limited intensity and penetration capacity of CLI prohibits its *in vivo* application, especially in the clinical setting. Although the enhancement effect of the dual-excitation-based strategy proposed in the current study cannot be used for all *in vivo* applications, it shows potential for use in the following fields. Firstly, in preclinical *in vivo* imaging, this strategy would make it possible to achieve whole-body imaging or even Cerenkov luminescence tomography (CLT) on mice. According to the research on RENPs-based up-conversion luminescence imaging conducted by Chen et al., whole-body imaging of mice could be achieved with a penetration depth of 3.2 cm [37], indicating a reasonable potential of our dual-excitation-REMPs-based strategy to achieve whole-body imaging or even CLT in mice. Secondly, this strategy is also helpful in current clinical CLI technologies, including endoscopy [3,14] and thyroid imaging [38]. The mean thickness of the human gastrointestinal tract is 3-4 mm, and the thickness can increase to several centimeters under pathological conditions [39]. The mean thickness of the human nuchal skinfold is 5.2 mm [40], and the location of the thyroid gland can be much deeper depending on the thickness of subcutaneous tissue and the capsula glandular thyroidea. As a result, even if the enhancement of CL penetration is modest, from several millimeters to 1-2 centimeters, the sensitivity of CLI in the above clinical applications may be significantly improved while reducing the required radioactive dose. Moreover, the high magnetic moment of certain rare-earth ions, like Gd^3+^, renders REMPs potent contrast agents for magnetic resonance imaging (MRI). Thus, the radionuclides combined with REMPs will be a potential contrast agent for CLI/MRI multimodality imaging.

Although we clarified the feasibility of REMPs-based enhancement of CLI, some limitations remain unresolved. Firstly, in this proof-of-concept study, the images were taken upon physically mixing REMPs and radioactive sources in DMSO. The interactions between the REMPs and radioactive sources were unclear. The emission intensity may be affected by the distance and conjunction behaviors between the radionuclide and REMPs. Secondly, the CL energy varies with different radionuclides [7]; thus, other radionuclides should be explored to determine whether better REMPs-assisted CLI could be achieved. Thirdly, although the intensity and penetration of CLI has been enhanced by 2-fold, the extent of this enhancement by the addition of REMPs, especially on the intensity of CLI, was not as high as that achieved by QDs in previous studies [18,19]. There is still room for extensive improvement in the energy transfer efficiency of the REMPs to further enhance the intensity and penetration of CL. And according to the research on REMPs-based up-conversion luminescence imaging conducted by Liu et al., the REMPs could be modified and it is possible to delivery of the REMPs to the tissue of interest by coating the REMPs with PEG and labeled with radionuclides (for excitation). Therefore, further investigations are still needed to address these above limitations. 

## Conclusion

This study demonstrated that Er^3+^, Yb^3+^-doped REMPs can be dually excited by Cerenkov luminescence, producing overlaid emissions at 660 nm with improved intensity and penetration. The proposed strategy can significantly enhance the penetration and intensity of Cerenkov luminescence imaging, indicating its potential applications in the clinical settings.

## Supporting Information

Figure S1 **Relationship between the radioactivity and the quantity of REMPs.** (A) CLI of 3.7 MBq ^18^F-FDG with increasing quantities of REMPs (from 0 to 2.0 mg/mL). (B) Linear regression of the influence of the quantity of REMPs on the enhancement of Cerenkov luminescence intensity.
(TIF)

Figure S2 **Relationship between the enhanced luminescence intensity and radioactivity of the radionuclide.** (A) CLI of increasing ^18^F-FDG radioactivity from 0 to 3.7 MBq with 2 mg/mL of REMPs. (B) Linear regression of the influence of the radioactivity of ^18^F-FDG on the enhancement of Cerenkov luminescence intensity.
(TIF)

File S1 **Supplementary Methods.**
(DOCX)
