# Supplementary figures and images for: Enhancement of Cerenkov Luminescence Imaging by Dual Excitation of Er3+, Yb3+-Doped Rare-Earth Microparticles
Source: PLoS One. 2013 Oct 25;8(10):e77926. doi: 10.1371/journal.pone.0077926 (PMC3808356; doi:10.1371/journal.pone.0077926)

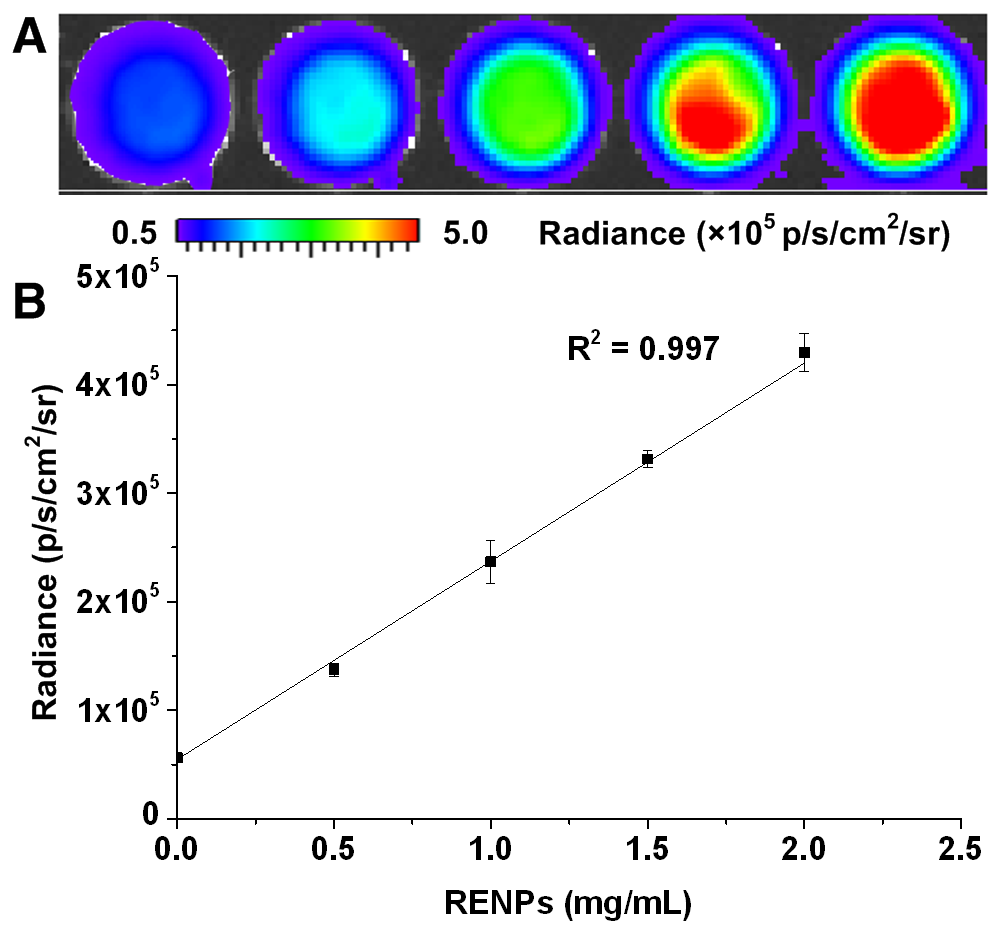

Supplement: Figure S1 — Relationship between the radioactivity and the quantity of REMPs. (A) CLI of 3.7 MBq 18F-FDG with increasing quantities of REMPs (from 0 to 2.0 mg/mL). (B) Linear regression of the influence of the quantity of REMPs on the enhancement of Cerenkov luminescence intensity. (TIF) [file pone.0077926.s001.tif]

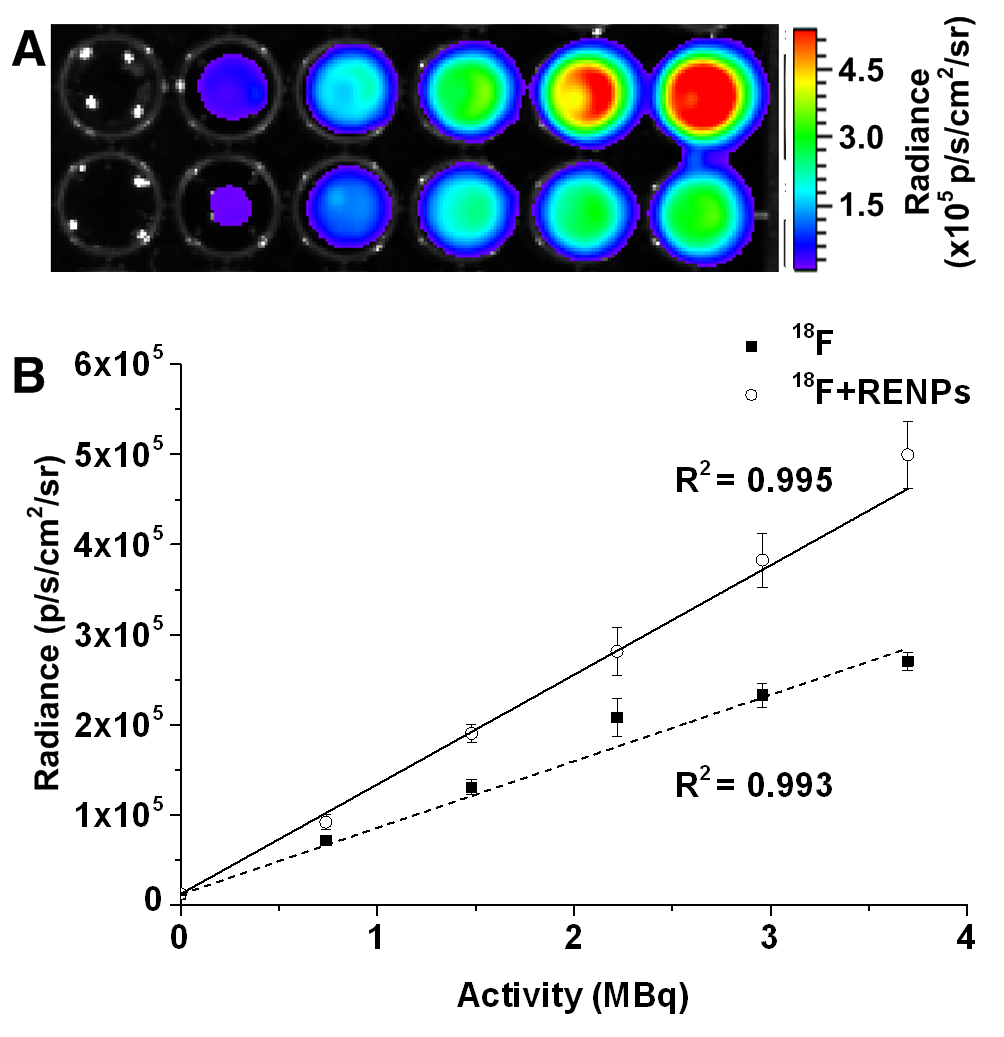

Supplement: Figure S2 — Relationship between the enhanced luminescence intensity and radioactivity of the radionuclide. (A) CLI of increasing 18F-FDG radioactivity from 0 to 3.7 MBq with 2 mg/mL of REMPs. (B) Linear regression of the influence of the radioactivity of 18F-FDG on the enhancement of Cerenkov luminescence intensity. (TIF) [file pone.0077926.s002.tif]
