# Supplementary material for: Enhancement of Cerenkov Luminescence Imaging by Dual Excitation of Er3+, Yb3+-Doped Rare-Earth Microparticles
Source: PLoS One. 2013 Oct 25;8(10):e77926. doi: 10.1371/journal.pone.0077926 (PMC3808356; doi:10.1371/journal.pone.0077926)
Supplement: File S1 — Supplementary Methods. (DOCX) [file pone.0077926.s003.docx]

The influence of the radioactivity and the quantity of REMPs on the enhancement of luminescence intensity was also analyzed. We chose ^18^F-FDG as an example because of the similar spectral characteristics of ^18^F-FDG and Na^131^I. A series of samples were prepared in 96-well black plates containing REMPs at concentrations of 0, 0.5, 1.0, 1.5, or 2.0 mg/mL dissolved in DMSO and 3.7 MBq ^18^F-FDG, with a final volume of 200 μL. The relationship between the concentration of REMPs and the luminescence intensity (Figure S1A). The luminescence intensity was linearly positively correlated with the concentration of REMPs (*R^2^* = 0.995; Figure S1B).

To investigate the relationship between the enhanced luminescence intensity and radioactivity of the radionuclide, 2 groups of samples were prepared in 96-well black plates with ^18^F-FDG at concentrations of 0, 0.7, 1.4, 2.2, 2.9, or 3.7 MBq with or without 2 mg/mL REMPs in each well, with a final volume of 200 μL. Total luminescence intensity was then measured. When the REMPs concentration was fixed (2 mg/mL), the detected emission intensity was linearly positively correlated with the radioactivity of ^18^F-FDG (*R*^2^ = 0.997). It should be noted that slope of the ^18^F-FDG+REMPs group was higher than that of ^18^F-FDG alone (Figure S2A and S2B).
